# Effect of Comprehensive Oncogenetics Training Interventions for General Practitioners, Evaluated at Multiple Performance Levels

**DOI:** 10.1371/journal.pone.0122648

**Published:** 2015-04-02

**Authors:** Elisa J. F. Houwink, Arno M. M. Muijtjens, Sarah R. van Teeffelen, Lidewij Henneman, Jan Joost Rethans, Florijn Jacobi, Liesbeth van der Jagt, Irina Stirbu, Scheltus J. van Luijk, Connie T. R. M. Stumpel, Hanne E. Meijers-Heijboer, Cees van der Vleuten, Martina C. Cornel, Geert Jan Dinant

**Affiliations:** 1 Department of Clinical Genetics, Section Community Genetics, EMGO Institute for Health and Care Research, VU University Medical Center, Amsterdam, The Netherlands; 2 School for Public Health and Primary Care, Department of Family Medicine, Maastricht University, Maastricht, The Netherlands; 3 Department of Educational Development and Research, Faculty of Health, Medicine and Life Sciences, Maastricht University, Maastricht, The Netherlands; 4 Skillslab, Faculty of Health, Medicine & Life Sciences, Maastricht University, Maastricht, The Netherlands; 5 Dutch College of General practitioners (NHG), Utrecht, The Netherlands; 6 Centre for Infectious Disease Control, National Institute for Public Health and the Environment, Bilthoven, The Netherlands; 7 Department of Resident Training, Maastricht University Medical Centre +, Maastricht, The Netherlands; 8 Department of Clinical Genetics, School for Oncology and Developmental Biology, Maastricht UMC+, Maastricht, The Netherlands; 9 Department of Clinical Genetics, VU University Medical Center, Amsterdam, The Netherlands; Cancer Research Centre of Lyon, FRANCE

## Abstract

General practitioners (GPs) are increasingly called upon to identify patients at risk for hereditary cancers, and their genetic competencies need to be enhanced. This article gives an overview of a research project on how to build effective educational modules on genetics, assessed by randomized controlled trials (RCTs), reflecting the prioritized educational needs of primary care physicians. It also reports on an ongoing study to investigate long-term increase in genetic consultation skills (1-year follow-up) and interest in and satisfaction with a supportive website on genetics among GPs. Three oncogenetics modules were developed: an online Continuing Professional Development (G-eCPD) module, a live genetic CPD module, and a “GP and genetics” website (*huisartsengenetica*.*nl*) providing further genetics information applicable in daily practice. Three assessments to evaluate the effectiveness (1-year follow-up) of the oncogenetic modules were designed: 1.An online questionnaire on self-reported genetic competencies and changes in referral behaviour, 2.Referral rates from GPs to clinical genetics centres and 3.Satisfaction questionnaire and visitor count analytics of supportive genetics website. The setting was Primary care in the Netherlands and three groups of study participants were included in the reported studies:. Assessment 1. 168 GPs responded to an email invitation and were randomly assigned to an intervention or control group, evaluating the G-eCPD module (n = 80) or the live module (n = 88). Assessment 2. Referral rates by GPs were requested from the clinical genetics centres, in the northern and southern parts of the Netherlands (Amsterdam and Maastricht), for the two years before (2010 [n = 2510] and 2011 [n = 2940]) and the year after (2012 [n = 2875]) launch of the oncogenetics CPD modules and the website. Assessment 3. Participants of the website evaluation were all recruited online. When they visited the website during the month of February 2013, a pop-up invitation came up. Of the 1350 unique visitors that month, only 38 completed the online questionnaire. Main outcomes measure showed long-term (self-reported) genetic consultation skills (i.e. increased genetics awareness and referrals to clinical genetics centres) among GPs who participated in the oncogenetic training course, and interest in and satisfaction with the supportive website. 42 GPs (52%) who previously participated in the G-eCPD evaluation study and 50 GPs (57%) who participated in the live training programme responded to the online questionnaire on long-term effects of educational outcome. Previous RCTs showed that the genetics CPD modules achieved sustained improvement of oncogenetic knowledge and consultation skills (3-months follow-up). Participants of these RCTs reported being more aware of genetic problems long term; this was reported by 29 GPs (69%) and 46 GPs (92%) participating in the G-eCPD and live module evaluation studies, respectively (Chisquare test, p<0.005). One year later, 68% of the respondents attending the live training reported that they more frequently referred patients to the clinical genetics centres, compared to 29% of those who attended the online oncogenetics training (Chisquare test, p<0.0005). However, the clinical genetics centres reported no significant change in referral numbers one year after the training. Website visitor numbers increased, as did satisfaction, reflected in a 7.7 and 8.1 (out of 10) global rating of the website (by G-eCPD and live module participants, respectively). The page most often consulted was “family tree drawing”. Self-perceived genetic consultation skills increased long-term and GPs were interested in and satisfied with the supportive website. Further studies are necessary to see whether the oncogenetics CPD modules result in more efficient referral. The results presented suggest we have provided a flexible and effective framework to meet the need for effective educational programmes for non-geneticist healthcare providers, enabling improvement of genetic medical care.

## Introduction

Innovative developments in genetics are increasingly becoming applicable in routine medical care. As a result, general practitioners (GPs) are confronted with challenging genetic information, patients’ requests for genetic tests and their diagnostic and therapeutic consequences. Successful implementation of genetic innovations requires overcoming several barriers, including the fact that physicians lack knowledge of genetics relevant for daily practice, are insufficiently aware of options for genetic testing, and report being insufficiently able to deliver genetic services [1,2]. If genetics is to have an effect on clinical practice comparable to its impact on research, health-care providers will need to improve their genetic literacy [3].

Physicians generally prefer to be educated in a practical manner, which means genetics education should be applicable in daily practice, using case-based learning [4–6]. Genetic core competencies for non-geneticist health care workers have been formulated [7–10]. Competencies are needed in three domains: cognitive (knowledge), psychomotor (skills) and affective (attitude) [11]. Combining the educational competencies in training courses is assumed to have greater impact on clinical performance than training the competencies separately [12].

We set out to provide a flexible and effective framework for genetics education for primary care physicians, based on multiple educational methods and assessable at the highest possible level of evaluating the learning process and its effects on genetic performance in daily practice. Gaff’s model taught us how to work towards an effective genetics education programme. This model describes the development and evaluation of genetics education programmes and is informed by three theories: *adult learning theory*, *programme logic modelling and evaluation theory* [13].

Our project started by exploring the needs for and role of genetics in primary care and identified priorities in genetic education as mentioned by GPs [5,6]. The top three genetic competencies were “Recognizing signals that can indicate a hereditary component of a disease”, “Evaluating indications for referral to a clinical genetics centre”, and “Knowledge of the possibilities and limitations of genetic tests” [6]. We expected that training focusing on these topics would lead to higher quality consultations between medical professionals and patients, reflected in timely referrals to the specialized departments of clinical genetics. The genetic competencies we studied related to oncology, as this was that year’s focal theme of the Dutch College of General practitioners (NHG).

Based on the priorities, and integrating genetic core competences, we developed three training modules implemented by the NHG (Department of Education):

1. an online continuing professional development module on oncogenetics (G-eCPD) [14],
2. a live genetic CPD module (interactive programme taking oncogenetics as a model condition) [15] and
3. a supportive website (www.huisartsengenetica.nl, “*GP and genetics”)* [14,15].

An online evaluation study showed a significant improvement in oncogenetic knowledge (G-eCPD) and consultation skills (live module) at follow-up after the interventions. Satisfaction and self-reported competence in daily practice were high for both training modules [14,15].

The website was developed and is being kept up-to-date by the research team in collaboration with the Erfocentrum (a Dutch information centre on heredity and genetic disorders supported by the Ministry of Health) and NHG, with on-demand supportive information enabling users to work on the learning tasks required from the two learning modules and apply genetic competencies in routine general practice. The easily accessible website provides GPs with on-demand information on e.g. basic genetics and referral to clinical genetics centres.

To our knowledge, this was the first time such a set of oncogenetic training modules has been organized and evaluated based on prioritized topics and effects on genetic performance.

### Kirkpatrick’s framework for evaluating educational outcomes

**Fig 1:**
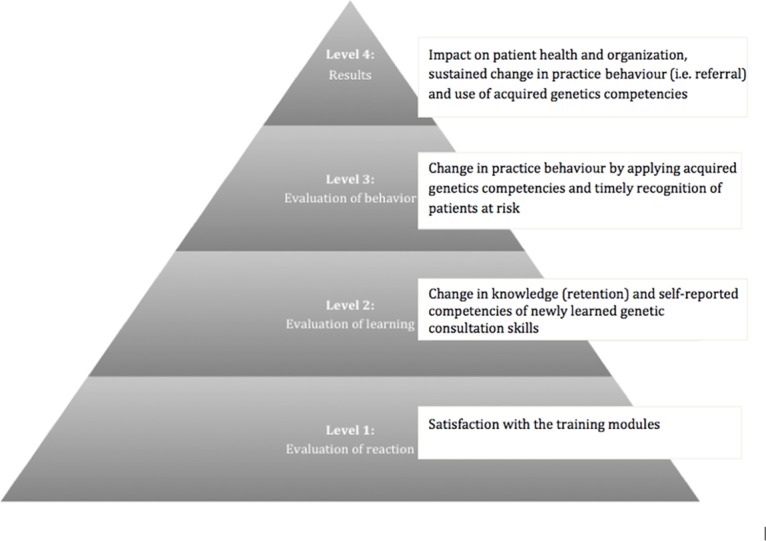
Genetics educational framework. Based on Kirkpatrick’s Evaluation Framework for Educational Outcomes [16,18]

For our effect evaluation we used Kirkpatrick’s framework for evaluating educational outcomes, originally presented in 1967 and distinguishing four levels: valuation (level 1; satisfaction), learning (level 2; knowledge and knowledge retention), behaviour (level 3: applying knowledge and consultation skills regarding timely recognition of patients at risk) and effects on patient health and organization (level 4: changes in actual practice performance [i.e. referral] and results) [16]. The impact on society, or patient safety in genetic medical care, is part of level 4 (Fig 1). We used Moore’s model of CPD curriculum design, identifying individual learning steps with their educational objectives and using the Kirkpatrick framework as a model to evaluate our oncogenetic modules [17]. Table 1 shows a combined overview of these models of CPD curriculum design and evaluation of learning outcomes, at four consecutive levels. The first learning step according to Moore would be to try and inform GPs and aim for a better understanding of oncogenetics. The first level of Kirkpatrick’s framework for evaluating the educational outcome then assesses satisfaction with the oncogenetic modules. The higher the level, the more complex the potential learning outcome of the oncogenetic module (up to the actual impact on health).

**Table 1:** Levels of oncogenetics training modules and evaluation according to Kirkpatrick and Moore (Adjusted according to Davis et al., 2008 [4]).

Kirkpatrick/Moore levels of education and evaluation	Kirkpatrick definition	Oncogenetics module format	Assessment	Educational objective
I	Satisfaction	G-eCPD [14], live module [15], supportive website	Satisfaction questionnaire and website visitor count	Information, understanding
II	Knowledge, self-reported competences of newly learned consultation skills	G-eCPD [14], live module [15]	Multiple-choice questions, open-ended questions, vignettes: pre/post and retention test	Information, understanding
III	Behavioural change	Live module [14]	Responses to SP encounters in actual practice: pre/post and retention test	Synthesis, application, performance, attitude
IV	Organizational change, health gain	G-eCPD [14], live module [15], supportive website	GP referral data from clinical genetics centres	Analysis, synthesis, evaluation: health gain through timely (increased) referral to clinical genetics centres

*G-eCPD*: *online continuing professional development on oncogenetics; GP*: *general practitioner; SP*: *standardized patient*

In our earlier publications we reported on the evaluation of levels 1 and 2 in Kirkpatrick’s hierarchy, showing increased knowledge and skills [14,15]. Here we aimed to answer the following research questions to try and address levels 3 and 4:

1. What are the long-term effects on genetic consultation skills among GPs participating in the training modules? We assessed self-reported skills (i.e. increased genetics awareness and referrals to clinical genetics centres), and compared GPs’ referral data to clinical genetics centres before and after training.
2. What is the interest in and satisfaction with the website? We analysed website visitor counts and determined satisfaction with the website by means of a pop-up questionnaire. Visitor count was expected to increase after the introduction of the oncogenetics programmes and as a result of increased media attention such as links on social media, newsletters and media newsflashes.

## Materials and Methods

Four instruments were used to answer the above research questions:

1. An online questionnaire determining self-reported genetic consultation skills was emailed to those who had previously attended one of the oncogenetics CPD modules one year earlier. See the online-only materials for background details for the Materials section (“Questionnaire to determine self-reported applicability of an online continuing professional development (G-eCPD) module and a live training module”).
2. Referral rates by GPs were requested from the clinical genetics centres in the northern and southern parts of the Netherlands (respectively VU University Medical Centre [VUMC], Amsterdam and Maastricht University Medical Centre [MUMC], Maastricht) for the two years before (2010 and 2011) and the year after (2012) the launch of our oncogenetics CPD modules and the website. Changes in referral rates were assumed to estimate GPs’ synthesis and application of the newly learned oncogenetics knowledge and consultation skills, and increased awareness of oncogenetics problems in daily practice.
3. Website visitor analytics roughly determined the GPs’ sustained interest in the supportive website one year after its introduction, suggesting changes in practice reorganization and consequently health gains. Visitor count was expected to go up after the introduction of the oncogenetics programmes and as a result of increased media attention, such as links on social media, newsletters and media newsflashes.
4. Interest in and satisfaction with the website were investigated more specifically with the help of an online pop-up questionnaire, which was available for one month when visitors visited the website. See the online-only materials for background details for the Materials section (“Website satisfaction questionnaire”).

The medical ethics committees of the Netherlands Association for Medical Education (NVMO), Maastricht University Medical Centre (MUMC+), and VU University Medical Center Amsterdam (VUMC), The Netherlands, approved the study protocols. All participants gave written informed consent before the trials.

### Participants

The project team collaborated with the NHG in providing CPD modules on genetics [14,15]. The GPs who had previously participated in the accompanying evaluation studies and were working full-time or part-time in family practice, were all followed up in the longer term as they participated in the online questionnaires. Recruitment for participation in the online questionnaire to evaluate the live oncogenetics CPD module was limited to GPs practicing in two Dutch provinces where the previously held live training sessions had been given (n = 88). Participants for the online oncogenetics training were recruited outside these two provinces, to be able to assess effects separately (n = 80).

Participants of the website evaluation were all recruited online, through a pop-up invitation that would come up when they visited the website during the month of February 2013.

### Analysis

The answers to the online questionnaire (effects of online and live training) and the pop-up questionnaire (appreciation of the website) were analysed in similar ways. To facilitate interpretation, the 5-point scales were converted to 2-point (binomial) scales by merging the lower three categories (disagree completely, disagree, neither disagree nor agree) and the upper two (agree, agree completely). The proportion of answers in the upper category of the binomial scale and the associated 95% confidence interval were calculated to assess the effect of the training as reported by the GPs, and the appreciation of the website by its visitors.

Website visitor counts were obtained by Google Analytics for the September 2011 to March 2013 period. Time series were obtained for the number of visits per month, the number of pages viewed per month, and the number of pages per visit per month. Where relevant, the mean trend in the time series was estimated by fitting a straight line (linear regression) to the data, and using the slope of the line as an indicator of trend.

Data were analysed using SPSS20.

## Results

### GPs’ self-reported skills and referral

#### Participant characteristics

Forty-two GPs (52%) who had participated in the G-eCPD evaluation study and 50 GPs (57%) who had participated in the live training program responded to the online questionnaire on long-term effects at Kirkpatrick’s second level of educational outcome evaluation. Eighty-eight percent of the respondents who had attended the live training reported that they now more frequently considered referring patients to the clinical genetics centres, compared to 64% of the respondents who had attended the online CPD module (Table 2, Chisquare test, p<0.01). Sixty-eight percent and 29%, respectively, reported that they actually referred patients more frequently (Chisquare test, p<0.0005).

**Table 2:** Self-reported applicability of an online continuing professional development (G-eCPD) module and a live training module on oncogenetics, by GPs who participated in one of these CPD modules.

Statement/Question	Response	Online continuing professional development, module (G-eCPD)				Live interactive training programme on oncogenetics				Significance of the between-group difference
	Category	Total number of respondents	%	95%-CI		Total number of respondents	%	95%-CI		p
				lo	hi			lo	hi	
I am more aware of genetic problems	Agree, Agree completely	42	***69***	53	82	50	***92***	81	98	***0*.*0047***
I have treated more patients with genetic problems	Agree, Agree completely	42	***12***	4	26	50	***46***	32	61	***0*.*0004***
I have more frequently considered referring patients to the Clinical Genetics Department	Agree, Agree completely	42	***64***	48	79	50	***88***	76	96	***0*.*0069***
I have more frequently referred patients to the Clinical Genetics Department	Agree, Agree completely	42	***29***	16	45	50	***68***	53	81	***0*.*0002***
I am better able to explain possibilities/limitations of genetic tests to patients	Agree, Agree completely	42	***50***	34	66	50	***72***	58	84	***0*.*0304***
How frequently do you use the genetics website?	Once to Daily	38	***18***	8	34	47	***47***	32	62	***0*.*0061***
Will you keep on using the genetics website?	Yes	31	81	63	93	25	96	80	100	0.0841
Did you ever consult the genetics website when referring patients to the Clinical Genetics Department?	Yes	7	71	29	96	22	91	71	99	0.1930
I would recommend the genetics website to my colleagues	Agree, Agree completely	7	86	42	100	22	91	71	99	0.6943
			Mean				Mean			
Global rating of the genetics website	10-point	7	7.7	7.0	8.4	22	8.1	7.8	8.3	0.1931

*95%-CI*: *95% confidence interval; G-eCPD*: *online continuing professional development on oncogenetics; GP*: *general practitioner; p*: *p-value for the Chi-square test for the between-group difference (Online CPD vs*. *Live training) of the percentage (Agree*,*Agree completely) in response to the statements*, *and independent-samples t-test for the between-group difference in the global rating (significant results indicated in italic and bold)*

#### Changes in referral to clinical genetics centres

Table 3 shows the results on GPs’ referral rates to clinical genetics centres, found by searching the ICT system of the clinical genetics centres in Amsterdam and Maastricht for the years 2010–2012. No significant changes in referral rates were seen in the year after the introduction of the oncogenetics modules and website.

**Table 3:** GP Referral rates to the clinical genetics centres in the northern and southern parts of the Netherlands for the years 2010–2012.

Site	Year		
	2010	2011	2012
Maastricht University Medical Centre	1549	1590	1508
VU University Medical Center Amsterdam	961	1350	1367
Total	2510	2940	2875

#### Website interest and satisfaction

**Fig 2:**
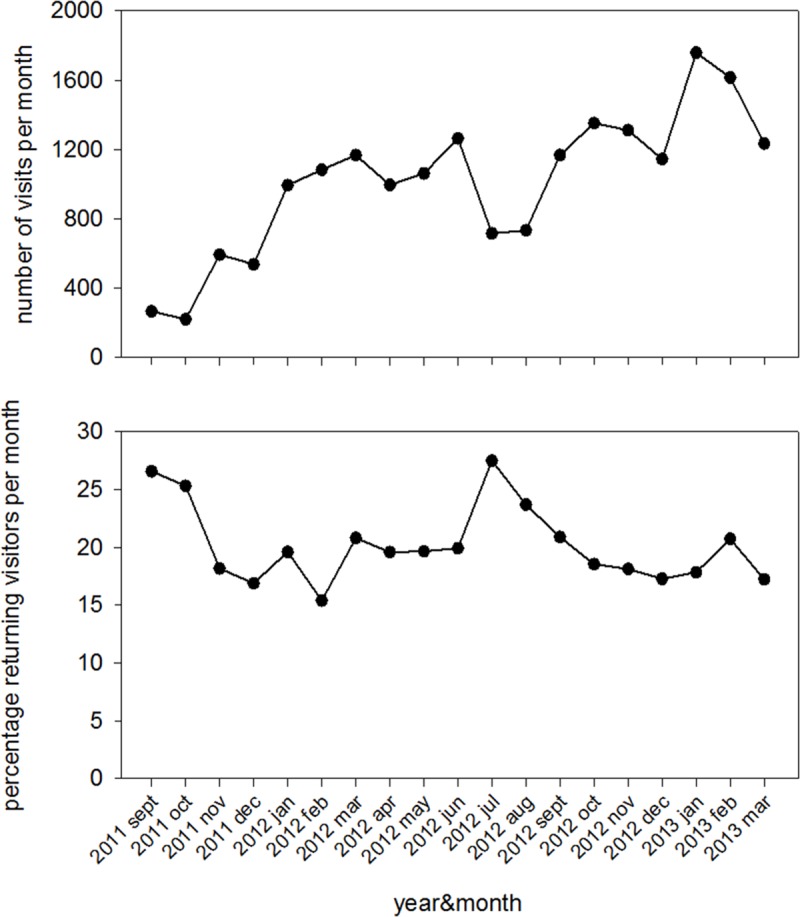
Website visitor numbers (Top figure) and percentage returning visitors per month.

Thirty-eight visitors (12 [32%] aged 31–40 years, 27 [71%] female) to the website completed the popup questionnaire (results of the questionnaire shown in Table 4). Fig 2 (upper panel) shows that website visitor numbers steadily increased, with almost 60 new visitors each month. The percentage of returning visitors (Fig 2) was stable at around 20% each month, demonstrating sustained interest in the website. Website visitor analytics showed a top 10 of most frequently visited pages within the site, which were drawing family trees, hereditary diseases, family history taking and consanguinity, and the desire to become pregnant. The results suggest increased use of genetic knowledge and consultation skills, conceivably reflecting a potential for improved patient genetic health.

**Table 4:** Self-reported satisfaction and applicability of the genetics website by general visitors and by GPs only.

Statement/Question	Response	All respondents				GPs only			
	Category	Total number of resp.	%	95%-CI		Total number of resp.	%	95%-CI	
				lo	hi			Lo	hi
Is this your first visit to the genetics website?	No	38	21	10	37	22	18	5	40
The content of the website is helpful	Agree, Agree completely	19	68	44	87	10	70	35	93
The content of the website is up to date	Agree, Agree completely	19	68	44	87	10	70	35	93
The content of the website is easy to understand	Agree, Agree completely	19	74	49	91	10	70	35	93
The content of the website lives up to my expectations	Agree, Agree completely	19	63	38	84	10	70	35	93
The content of the website is attractive	Agree, Agree completely	19	53	29	76	10	60	26	88
The content of the website is up to professional standards	Agree, Agree completely	19	74	49	91	10	80	44	98
The content of the website is clearly structured	Agree, Agree completely	19	63	38	84	10	70	35	93
The content of the website is simple to use	Agree, Agree completely	19	58	34	80	10	70	35	93
I would recommend the genetics website to my colleagues	Agree, Agree completely	19	68	44	87	10	80	44	98
Was your current visit successful?	Completely, or partly successful	19	84	60	97	10	100	69	100
			Mean				Mean		
Global rating of the genetics website	10-point	38	6.3	5.5	7.1	22	7.2	6.5	7.9

95%-CI: 95% confidence interval; *GP*: *general practitioner*

## Discussion

Our long-term evaluation of the effects of three CPD modules on oncogenetics for GPs showed that self-reported genetic consultation skills among the GPs had increased after one year of follow-up, including increased consideration of referral to clinical genetics centres. Regional GP referral numbers did not change in the year after the introduction of the modules.

An increase in the number of actual referrals was expected to reduce the number of missed cases [19]. A reason for this delay in referrals could be the limited number of GPs who participated in the oncogenetic CPD modules. Also, the CPD modules focused specifically on oncogenetics, so GPs’ referral rates might increase if the genetics CPD modules also addressed other topics such as cardiogenetics, diabetes or reproductive genetic counselling. GPs play a key role as gatekeepers to the Dutch health care system, when appropriate and timely referral to medical specialists is warranted. Their role could be enhanced, with increased awareness and alertness, by effectively educating more GPs [19]. Another possible explanation is that improved genetics knowledge and consultation skills do not necessarily result in more referrals. Improved knowledge and awareness could also lead to more accurate referrals and could consequently even lead to a decrease in number of referrals. Direct measurement of changes in referral rates or efficiency and effectiveness of referral was, however, impossible at this time, since no ICT tools are as yet in place to register family history in the Dutch Electronic Health Registry. This change in accuracy of referrals will need to be demonstrated through further studies in the near future.

Although only a small number of website visitors completed the pop-up questionnaire, those who did indicated they were interested in and satisfied with the supportive website. This was confirmed by the long-term website visitor counts and the percentage returning visitors, indicating sufficient website user-friendliness and usefulness. Website visitors often looked for information on basic genetics (drawing family trees, family history taking), while initially we had not expected a need for these topics. At the time, no website visitor analytics were available from comparable websites. Some website visitor analytics were available from websites with information intended for non-geneticist medical practitioners, the general public, patients, families and others. These, however, cannot be compared with the data provided on our supportive website, which mainly aims to inform GPs. With 8747 GPs actively working in the Netherlands, around 1800 visitors (presumably GPs) returning each month is promising. Updating the website is an ongoing process, which should sustainably increase website visitor numbers. Future studies should focus on improving the usefulness and user-friendliness of the website, and the website could perhaps be translated for GPs internationally, adapting the information to the specific circumstances in other countries where needed (such as specific information on referral and insurance). Whether all 1800 visitors each month are actually GPs is currently unknown and unverifiable without breaching confidentiality. Unpublished data from the pop-up questionnaire showed us, however, that 57.9% (n = 22) answered that they were GPs and 31.6% (n = 12) that they were patients. However, the response rate was small and these numbers can therefore only be regarded as indicative.

The study results suggest that we have provided an effective framework to answer the need for genetic education programmes for non-geneticist health-care providers. The training modules proved to represent a feasible and satisfactory method to achieve long-term improvement of useful oncogenetic knowledge and consultation skills. This educational framework can inform future training activities for GPs, and potentially also other medical practitioners, to enhance genetics-related consultation and decision-making, with the ultimate aim of improving medical care.

### Comparison with other studies

A meta-analysis, which examined the effect of moderator variables on physician knowledge, performance and patient outcomes, showed results similar to those of our study. It suggested a larger effect size in terms of CPD outcome in the case of interactive interventions, using multiple methods, designed for small groups of physicians from a single discipline [11]. However, the meta-analysis showed a negative correlation between effect size and the outcome assessment time (r = -0.31). This would imply that the longer the amount of time that elapses between a CPD intervention and the assessment of actual impact on daily practice could impair the retention of genetic competencies acquired. Longer-term outcome assessment of our oncogenetics training programme, regarding referral rates and changes in practice reorganization, would be required to allow anything to be said about the true impact on patients’ genetic health. Repeating the oncogenetics training programmes on a yearly basis for local GP groups might lead to better outcomes in the longer term. Effective new implementations of changes in patient care might change referral rates or improve the appropriateness of patient referral [20]. However, few rigorous evaluations of referral processes from primary to secondary care are available to base policy on [21]. A few promising interventions however, which we already incorporated in our live training module, were found to favourably influence the referral process, including local educational interventions involving secondary care specialists and structured referral sheets. This will need to be further studied for the case of referral to clinical genetics centres. Clear guidelines for referral should be distributed and should be part of the training courses. Genetic counsellors may also help the referral process, as they can provide a second opinion before a patient is referred to a specialist. Financial compensation to optimize referral has been studied, but there is insufficient evidence to draw firm conclusions [21].

### Methodological considerations

We used Gaff’s model to develop and evaluate an effective CPD programme on genetics education. The model is informed by three theories: *adult learning theory*, *programme logic modelling* and *evaluation theory* [13]. Briefly, *adult learning theory* proposes that for an education programme to be effective, it should be responsive and enable learners to optimize their learning. In previous studies we had identified a need among GPs to learn about genetics topics [5,6]. The first steps towards developing an effective genetics education programme involved the GP learners, focusing on content and process, and using a range of multifaceted teaching strategies, including experiential and interactive strategies.

*Programme logic modelling* is a useful theory to define and plan a programme design and possible evaluation, which inspired the Delphi study we held previously [6]. Experts helped us prioritize topics for a genetics education programme, based on prior focus group studies [5]. The experts suggested a top 10 of genetics topics that would be useful in routine GP practice, which could inform the further development of effective genetics education. Our approach of letting the health professionals participate in the development of the education programme by assessing their learning needs was intended to increase GPs’ awareness of the relevance of genetics in daily practice and to help them learn and apply the competencies acquired in daily genetic medical care.

The *evaluation theory* rigorously determines the impact of training programmes. Evaluations often show beneficial effects on process outcomes (e.g. increased genetic competencies), but evaluations of patient outcomes (e.g. improved genetic health care) often lag behind. Three concepts in this theory relating to genetic educational programme evaluation can be used to ultimately allow evaluation of patient outcomes. The first is *formative evaluation* (needs assessments by stakeholders involved). The second concept, *process evaluation*, relates to the manner in which a genetic educational programme is implemented in daily practice (i.e. family history taking and recording in GPs’ computerized information systems) and whether it reaches its intended audience in time (i.e. pre-symptomatic referral to the clinical geneticist). The last concept is *summative evaluation*, which sums up the impact of the programme on the users and audience involved (i.e. improved genetic competencies, awareness and behaviour of trained GPs, and genetic health-care outcome) [13]. The results of the summative evaluation show that applying the adult learning theory and programme logic model in our project, in assessing the needs and subsequently defining the programme design, was effective.

### Strengths and limitations

Voluntary participation by interested GPs may have caused selection bias. However, the baseline characteristics of the two groups in our previous RCTs were similar, and the participation rates (55% [G-eCPD] and 60% [live training] in the RCTs; 52% [G-eCPD] and 57% [live training] in the present study) were comparable to those in other studies among GPs (60%). This indicates that the participants were representative of the GPs who will likely attend oncogenetic training in the future [14,15,22,23]. It is possible that participating in the genetics training might become mandatory for all GPs. By applying an effective framework for genetics education and measuring the outcome of this educational programme at various levels in Kirkpatrick’s evaluation model, we were able to initiate a change in practice reorganization and identify barriers to implementation of genetics education [24]. Further assessments are necessary, however, to draw additional conclusions about patient health impacts. Rigorous assessment of appropriate referrals was not feasible within the project, as data were based on self-reported competencies, increases in referrals and website analytics, and referrals not registered in Electronic Patient Records (EPRs). We are planning to more rigorously assess changes in cancer-related referrals by GPs who participated in the RCTs to clinical genetics centres by looking at the number of referrals registered in the EPR using the ICPC codes added in the register [24]. Furthermore, to assess the actual user-friendliness and usefulness of our website, we plan to examine this using qualitative research methods.

## Conclusion

The results presented show that we have developed a flexible and effective framework for genetics education, which may possibly be applied for health care practitioners internationally. They also suggest that the website we developed supports the CPD training and daily practice of GPs.

The suggested training tools can guide the future development of curricula that are appropriate to the national context, educational system and health-care setting of the professionals involved. We conclude that it is possible to optimize genetic educational materials, as a multifaceted approach to meeting GPs’ needs for genetics education, prioritizing topics and improving core competencies taught in genetic education. This educational framework ultimately has the potential to improve the quality of genetic health care for patients.

Trial Registration: trialregister.nl Identifier: NTR3322 and NTR3323

## Supporting Information

S1 Online Only Text (DOCX)
