# Supplementary material for: Effect of Comprehensive Oncogenetics Training Interventions for General Practitioners, Evaluated at Multiple Performance Levels
Source: PLoS One. 2015 Apr 2;10(4):e0122648. doi: 10.1371/journal.pone.0122648 (PMC4383330; doi:10.1371/journal.pone.0122648)
Supplement: S1 Online Only Text — (DOCX) [file pone.0122648.s001.docx]

# Supporting Information document

# Online-only text

## Background details for the Materials section. Questionnaire to determine self-reported applicability of an online continuing professional development (G-eCPD) module and a live training module

An online questionnaire was emailed to those who had previously attended the oncogenetics CPD modules, to determine their long-term self reported genetic consultation skills (i.e. increased awareness of possible genetic predisposition for diseases, discussing potential familial and hereditary disease risks, managing potentially developing hereditary diseases, considering referral to clinical genetics centres more frequently and actually referring patients more frequently, and explaining the possibilities and limitations of oncogenetic testing). The questionnaire contained five items (with a 5-point Likert scale: 1=completely disagree; 5=completely agree) relating to different aspects of the self-reported applicability of genetic competencies.

## Background details for Materials section. Pop-up questionnaire assessing self-reported satisfaction and usefulness of the genetics website, by general visitors and by GPs.

The satisfaction questionnaire contained four items regarding the website content (helpful, recent information, easy to understand language, meeting expectations) and four on usefulness (attractiveness, professional, structure, easy to use) on a 5-point Likert scale (1=completely disagree; 5=completely agree), relating to different aspects of satisfaction.

Respondents were also asked to give a global rating of the quality of the website on a ten-point scale (1: useless; 5: insufficient 6: sufficient; 8: good 10: excellent), and about the frequency with which they used the website. The applicability questionnaire contained two 4-point scale items about the need for the supportive website and the value of the website for referral in daily practice, and a multiple-choice question about the pages respondents had used to inform referral to clinical genetics centres. There was also a question asking whether the participant would recommend the website to colleagues (yes/no).

The demographic survey asked about participants’ general characteristics (male/female; age; professional background (GP, midwife, medical specialist, medical student, patient/other).
